# County-to-county migration is associated with county-level racial bias in the United States

**DOI:** 10.1038/s41598-025-88218-7

**Published:** 2025-02-21

**Authors:** Rui Jin, Jimmy Calanchini, Kate A. Ratliff

**Affiliations:** 1 Department of Psychology, University of Florida, Gainesville, FL USA; 2 Department of Psychology, University of California Riverside, Riverside, CA USA

**Keywords:** Migration, Residential mobility, Prejudice, Regional bias, Human behaviour, Social evolution

## Abstract

Millions of people move within the U.S. each year. We propose that people function as proxies for their locations, bringing the culture of their previous residence to their new homes. As a result, migration might systematically influence regional biases across geographic units over time. Using county-to-county migration data from the U.S. census and county-level racial attitude estimates from Project Implicit, the present research examined the impact of people relocating from one U.S. county to another on racial attitudes in their new county. Consistent with our prediction, the bias brought by the migrants positively predicts county-level racial bias after migration, even after controlling for county-level racial bias before migration. This finding remains robust across various sample inclusion criteria and spans three time periods (2006–2010, 2011–2015, and 2016–2020). These results highlight the significant role of migration in spreading and shaping regional racial attitudes, emphasizing the importance of considering macro-societal processes such as migration when studying changes in regional racial attitudes.

Humans are constantly on the move. The number of international migrants—individuals residing in a country other than their birthplace—reached 281 million globally^1^. In a given year, about 15% of the U.S. population moves^2^. And, a growing proportion of people are relocating across geographic boundaries, such as to a different county or state^2^. Migration, often referred to as *residential mobility* in psychological research^3,4^, can profoundly influence the demographic and socioeconomic characteristics of a country, state, county, or other geographical area. Previous research suggests that migration might also influence the psychological characteristics of regions, including people’s racial attitudes (i.e., evaluations of racial groups as positive or negative)^5^. Consequently, the purpose of the present research is to understand how U.S. domestic migration over time influences county-level racial attitude in the United States.

## From micro to macro-perspective on residential mobility

Psychologists commonly study residential mobility to understand why people move and how the moving experience influences them personally. People often relocate for family and career reasons^6^, or to seek a better person-environment fit (e.g., to align with their ideological beliefs^7^). Personality traits also play a significant role in mobility, with people high in extraversion and openness more likely to relocate^8,9^. Both the experience of moving and living in regions with higher migration rates affect people in a variety of ways: they shape people’s self-concept and relationship with others^10,11^, they make people less risk-aversive and more open to new experiences^10^, but they may also adversely impact subjective well-being^11,12^.

The majority of scholarship on residential mobility primarily focuses on individual-level decisions, adjustments, and personal outcomes^13^. However, at the macro level, migration also affects socioeconomic and policy transformations across geographical units. The structure and magnitude of migration have been shown to explain collective psychological phenomena and their geographical distribution. For example, Buttrick and Oishi^14^ posit that the declining U.S. migration rate indicates a shift in cultural values at national level. State-level differences in mobility are related to regional differences in ethnocentrism and interest in other cultures^15^, tightness-looseness of the local culture^16^, and pro-community action^17^. These findings suggest that migration can shape the attitudes, norms, and behaviors of a region. Rentfrow and colleagues^5^ proposed migration as a mechanism in the formation of regional psychology, including racial attitudes. Yet no study that we know of has empirically tested migration as a mechanism for changing regional racial attitudes. Dovetailing with the arguments of Rentfrow and colleagues^5^, we propose that the infusion of values introduced by new residents (i.e., movers) can alter a region’s psychological characteristics. More specifically, we focus on how the racial attitudes of in-movers may shape regional racial attitudes over time.

## Regional racial attitude

Research on regional attitudes is flourishing due to theoretical advances and the availability of large-scale datasets such as those from Project Implicit^18,19^. Two prominent models have emerged to conceptualize regional attitudes as reflections of contextualized stereotypes. The Prejudice-in-Places model emphasizes how certain areas are characterized by predictable inequalities that systematically disadvantage specific social groups^20,21^. The Bias of the Crowds model^22^ further advanced theory, defining regional racial bias (used synonymously with *regional racial attitudes*) as a cognitive manifestation of the systemic racism of a place ^23^. Systemic racism, characterized by entrenched structural, institutional, and cultural patterns, reflects specific environmental conditions that perpetuate racial biases^24^. Thus, the bias of a region should be positively associated with outcomes that reinforce racial inequality. Indeed, through aggregating resident’s individual bias scores based on geographic proximity, researchers have correlated regional racial biases with a wide variety of important real-world outcomes, including Black-White disparities in traffic stop rates^25,26^, police militarization^27^, and the adoption rate of foster children^28^.

These aforementioned models suggest that regional racial biases (i.e., attitudes) have been built into local institutions within regions, and thus may be slower and more difficult to change than individual bias^29^. But what might prompt this (slow) change over time? Most regional research to date has been cross-sectional, leaving this question largely open and unanswered. At the national level, from 2007 to 2020, self-reported (i.e., explicit) bias favoring White relative to Black Americans in the U.S. decreased by 98% and the bias measured with the Implicit Association Test (i.e., implicit) by 26%. At the state level, antigay bias decreased at a sharper rate following local same-sex marriage legalization^30^. Both implicit and explicit regional biases also vary significantly across geographic units. Smaller geographic units (e.g., counties) have substantially more year-to-year variability in bias than do larger geographic units (e.g., states)^29^. Though some of this variability may be due to relatively more measurement error in smaller samples^31^, fluctuations in regional racial bias could also be explained by constant regional demographic changes, such as migration. Nevertheless, the macro-level factors that give rise to enduring attitude change within places^32^ remain poorly understood. The current research is the first to examine a macro-societal process—migration—on collective racial attitude change over time.

## Study overview

In the present research, we estimate regional attitudes by aggregating the attitudes of respondents who share geographic proximity. By knowing the regional racial bias of the place that people migrate *from*, we can infer the structural, institutional, and cultural patterns of their previous counties of residence. We propose people act as proxies for their regional culture. Thus, collectively, their bias should at least partially reflect the regional bias of the place they lived in. That is, when people move from one region to another, they bring the bias of their previous location with them. Importantly, this mechanism does not preclude the possibility that individual biases change when they move to another region and interact with their new neighbors, as proposed by Payne et al.^22^ and Smith & Conrey^33^. However, to date, existing evidence suggests that individual biases do not change significantly across the geographical boundaries a few months after somebody moves^34^.

To explore the impact of bias introduced by movers on a county’s regional bias over time, we integrated county-level migration flow data from the U.S. Census Bureau with county-level racial attitude data from Project Implicit. We hypothesized that movers function as proxies: to the extent that movers bring their biases from previous counties, then their migration should impact the biases of new counties in predictable ways. To test these idea, we conducted two sets of analyses, repeated over three time periods, to investigate: (a) whether the bias brought by movers predicts county-level bias after migration, above and beyond the baseline bias before migration; and (b) whether this effect is moderated by the scale of migration. We operationalized racial bias in two forms: implicit and explicit. Whereas considerable evidence indicates that implicit and explicit bias are distinct constructs at the individual level^35,36^, relatively more recent work has demonstrated that regional aggregates of implicit and explicit bias can correlate highly^29,30,37^. Consequently, we modeled regional implicit and explicit bias separately as an opportunity to conceptually replicate our findings across measurement methods.

## Methods

### Data sources

#### County-level migration

We obtained three five-year estimates of county-to-county migration from the United States Census Bureau^38^: 2006–2010, 2011–2015, and 2016–2020. The Census Bureau has released county-level migration estimates every five years since 2005, and the most recent release is 2016–2020. Thus, the three datasets we obtained contain all the estimates available except 2005. These data were based on the American Community Survey, which collects a series of monthly samples to produce migration estimates. Each dataset contains migration estimates of 50 U.S. states and Washington, D.C; each row of data represents the migration between a county pair (County A and B) where County A is the mover’s current residence and county B is the mover’s previous residence. In total, 52,205,933 people moved from one U.S. county to another between January 1, 2006 and December 31, 2020.

#### County-level implicit and explicit racial bias

We obtained responses on two measures of racial bias (a preference for White relative to Black Americans) from the Project Implicit Demo website (http://implicit.harvard.edu)^19^ between 2005 and 2021—the Implicit Association Test (IAT)^39^ and a self-reported preference. Previous research has shown explicit and implicit measures of racial bias are highly correlated at the regional level and that aggregated regional measures may capture a geographically meaningful construct that is not simply a result of measurement error canceling out^29,37^. Consistently, our data shows a strong correlation between county-level implicit and explicit bias (*r* = [0.56–0.60]). Therefore, in this study, we include the two bias measures at county-level as conceptual replications of one another.

The IAT assesses associations between two concepts (e.g., Black people and White people) and two attributes (e.g., good and bad). Exemplars representing each of the categories appear in the center of the computer screen and participants categorize them into one of the four superordinate categories as quickly as possible using two computer keys. Categorizing the exemplars more quickly when Black people and bad words (and White people and good words) share a response key compared to when Black people and good words (and White people and bad words) share a response key indicates an implicit preference for White people compared to Black people.

Explicit racial bias was measured by participants self-reported racial preference on a scale ranging from 1 = *I strongly prefer African Americans to European Americans* to 7 = *I strongly prefer European Americans to African Americans*).

To estimate the county-level racial bias, we calculate the average IAT *D* score for implicit bias and the average of self-report on racial preference for explicit bias. See Table 1 for descriptive statistics. As of now, no clear consensus exists on the minimum number of respondents in a county needed to accurately estimate the county-level racial bias (though see Calanchini et al.^37^ for some informal suggestions). Past research has applied arbitrary thresholds (e.g., *N* = 172 in Rosenbusch et al^40^; *N* = 1 in Snyder and Henry^41^). In this pre-registered study, we first examined racial bias of counties in which data is available from at least *N* = 20 participants. Then, to assess the robustness of our findings across participant thresholds, we employed multiple inclusion criteria (*N* = 1, 50, 100).

**Table 1:** Average implicit and explicit racial biases across U.S. counties with at least 20 respondents. Implicit bias was measured in *D* score, and explicit bias was measured with 7-point self-report. For both measures, higher scores indicate more anti-Black/pro-White bias.

Year	*N*	Implicit		Explicit	
		*M*	*SD*	*M*	*SD*
2006–2010	712	0.345	0.416	4.371	1.032
2011–2015	889	0.330	0.418	4.336	0.972
2016–2020	971	0.314	0.415	4.170	0.861

### Predictor variables

#### Aggregated move-in bias

To assess the attitudes brought into a county by people immigrating into it, we computed an *aggregated move-in bias* as follow:

$$\text{Move-in Bias }{A}_{i}=\frac{{\sum }_{j=1}^{i}\left(\text{Racial Bias }{B}_{ij}\times {N}_{ij}\right)}{{\sum }_{j=1}^{i}{N}_{ij}}$$

For any given County *A*, we compute the sum of regional racial bias (separately for implicit and explicit bias) brought by movers from each previous county (*B*_1_, *B*_2_… *B*_j_) weighted by the number of movers from that county (*N*_1_, *N*_2_…*N*_j_), then divided by the total number of movers from all previous counties *B*_1_ to *B*_j_. The computed move-in bias thus represents the aggregated bias brought by the movers to County *A*.

#### Migration flow

For any given County *A*, migration flow is the number of people who move in divided by its total population at the 5th year in each 5-year sample, expressed as a percentage. Census data provides the estimated number of people who moved from each County *B* to *A*. We sum them into a total number of movers and then use the 5th year total population of County *A* to convert the number into percentage. As pre-registered, we excluded counties with ± 2 *SD* of the average county-to-county migration flow. Counties experiencing exceptionally high or low migration flows may be influenced by unique economic, social, and policy factors, potentially distorting the overall results.

### Control variable

#### Baseline bias

Baseline bias is County *A*’s aggregated racial bias from the year before the 5-year period (i.e., county bias before the migration). Therefore, 2015 bias is the baseline bias for 2016–2020; 2010 bias is the baseline bias for 2011–2015; 2005 bias is the baseline bias for 2006–2010. We include baseline bias as a control variable so that we can isolate the effect of existing bias from the move-in bias in predicting the outcome bias.

### Outcome variable

#### Outcome bias

Outcome bias represents County *A*'s aggregated racial bias from the year immediately after the five-year period (i.e., county bias after the migration). Therefore, 2021 bias is the outcome bias for 2016–2020; 2016 bias is the outcome bias for 2011–2015; 2011 bias is the outcome bias for 2006–2010.

### Hypotheses and analysis plan

To the extent that movers bring their biases with them, we expect aggregated move-in bias to be positively correlated with county-level outcome bias, controlling for baseline bias. Furthermore, we anticipate that counties with higher migration flow will show a stronger correlation between move-in bias and outcome bias, controlling for baseline bias. The impact of bias introduced by movers is likely to be more pronounced in counties with a larger proportion of movers. To test these hypotheses, we conducted two linear regressions with the following specifications: (1) Model 1: Outcome bias = Aggregated move-in bias + Baseline bias; (2) Model 2: Outcome bias = Aggregated move-in bias * Migration flow + Baseline bias. We ran each analysis separately on county-level implicit and explicit racial bias, for a total of four analyses per 5-year sample. Aggregated move-in bias and migration flow were centered to correct for multicollinearity (Aiken et al., 1991). Counties that are more geographically proximate may also more interdependent, which potentially violates independence assumptions that underpin linear regression. Consequently, we supplemented our primary analyses with methods to account for spatial dependence^42^. Specifically, we used longitude-latitude decimal degrees of county centroid as point coordinates and created spatial weights matrix with each county’s *k*-nearest neighbors (*k* = 4). We chose the distance-based matrix approach due to the dispersed distribution of counties in our data. We then calculated Moran’s *I* test to check the spatial autocorrelation of the residuals from the models. Because the results of analysis were not significant—indicating no spatial dependency—we present them in the supplementary materials and report the standard linear regression models here. All the pre-registered exploratory analyses are also included in the supplementary material.

## Results

### Aggregated move-in bias predicts outcome bias for both implicit and explicit bias

#### Implicit bias

**Fig. 1:**
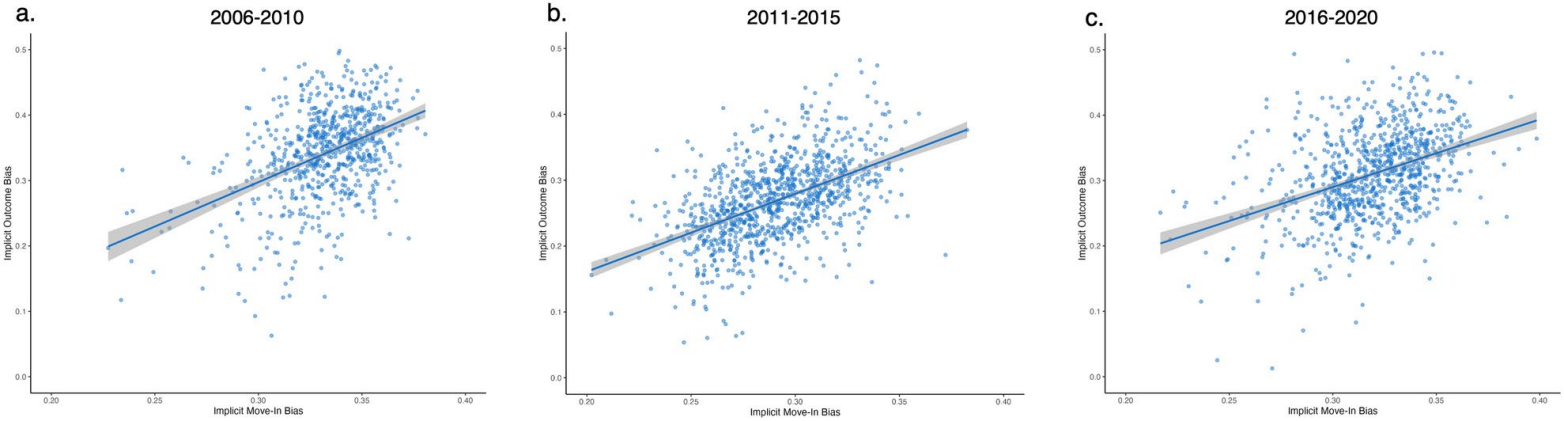
Observed and predicted implicit move-in bias as a function of county-level implicit outcome bias. More positive scores indicate higher anti-Black/pro-White bias. Shaded area represents 95% confidence intervals.

Aggregated move-in bias positively correlated with implicit outcome bias in all three datasets: 2006–2010 (β = 1.44, *t*(709) = 12.67, *p* < 0.001), 2011–2015 (β = 0.78, *t*(886) = 8.75, *p* < 0.001), and 2016–2020 (β = 1.01, *t*(968) = 15.04, *p* < 0.001). Additionally, and as expected, baseline implicit bias was positively correlated with the implicit outcome bias in all three datasets: 2006–2010: β = 0.19, *t*(709) = 5.16, *p* < 0.001; 2011–2015: β = 0.21, *t*(886) = 7.61, *p* < 0.001; 2016–2020: β = 0.22, *t*(968) = 9.24, *p* < 0.001. Across three 5-year periods, controlling for baseline bias, counties with more implicitly biased incoming movers became more implicitly biased after the migration (illustrated in Fig. 1a–c).

#### Explicit bias

**Fig. 2:**
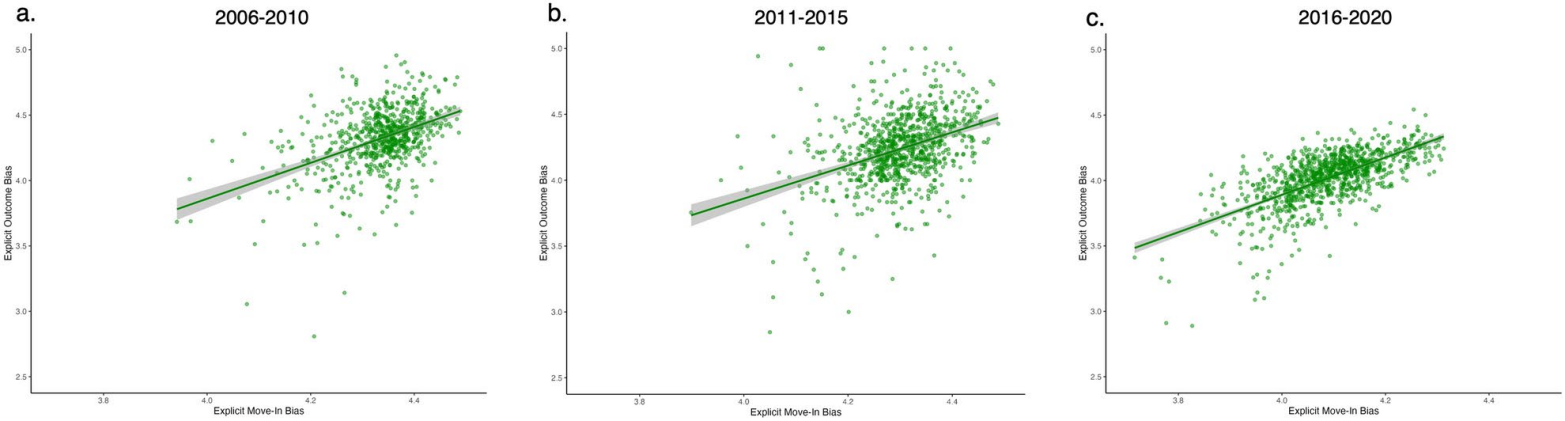
Observed and predicted explicit move-in bias as a function of county-level explicit outcome bias. More positive scores indicate higher anti-Black/pro-White bias. Shaded area represents 95% confidence internal.

 Aggregated move-in bias also positively correlated with explicit outcome bias in all three datasets: 2006–2010 (β = 1.16, *t*(709) = 11.22, *p* < 0.001), 2011–2015 (β = 0.69, *t*(886) = 6.14, *p* < 0.001), and 2016–2020 (β = 1.13, *t*(968) = 21.30, *p* < 0.001). Baseline explicit bias was again positively correlated with explicit outcome bias in all three datasets: 2006–2010: β = 0.29, *t*(709) = 7.76, *p* < 0.001; 2011–2015: β = 0.39, *t*(886) = 11.55, *p* < 0.001; 2016–2020: β = 0.34, *t*(968) = 13.69, *p* < 0.001. Similar to the results for implicit bias, across three 5-year periods and controlling for baseline bias, counties with more explicitly biased incoming movers became more explicitly biased after the migration (illustrated in Fig. 2a–c). All other details can be found in Model 1 of Tables 2 and 3.

**Table 2:** Model results for implicit racial bias. Model 1: Outcome bias = Aggregated move-in bias + Baseline bias. Model 2: Outcome bias = Aggregated move-in bias * Migration flow + Baseline bias.

		Predictor	*B*	β	*SE*	*t*	*p*
2006–2010	Model 1	Move-In Bias	0.425	1.444	0.114	12.668	< .001
		Baseline Bias	0.173	0.194	0.038	5.155	< .001
	Model 2	Move-In Bias	0.411	1.395	0.116	11.994	< .001
		Baseline Bias	0.176	0.197	0.038	5.244	< .001
		Flow	0.016	0.001	0.001	0.491	.623
		Move-In * Flow	0.123	0.138	0.051	2.679	.008
2011–2015	Model 1	Move-In Bias	0.293	0.776	0.089	8.753	< .001
		Baseline Bias	0.255	0.207	0.028	7.613	< .001
	Model 2	Move-In Bias	0.286	0.759	0.089	8.535	< .001
		Baseline Bias	0.256	0.209	0.027	7.685	< .001
		Flow	-0.076	− 0.003	0.001	− 2.541	.011
		Move-In * Flow	-0.051	− 0.044	− 0.942	0.347	.347
2016–2020	Model 1	Move-In Bias	0.425	1.009	0.067	15.035	< .001
		Baseline Bias	0.261	0.219	0.024	9.238	< .001
	Model 2	Move-In Bias	0.419	0.995	0.067	14.793	< .001
		Baseline Bias	0.255	0.214	0.024	9.000	< .001
		Flow	− 0.063	− 0.002	0.001	− 2.364	.018
		Move-In * Flow	− 0.017	-0.014	0.033	− 0.425	.671

**Table 3:** Model results for explicit racial bias. Model 1: Outcome bias = Aggregated move-in bias + Baseline bias. Model 2: Outcome bias = Aggregated move-in bias * Migration flow + Baseline bias.

		Predictor	*B*	β	*SE*	*t*	*p*
2006–2010	Model 1	Move-In Bias	0.375	1.159	0.103	11.224	< .001
		Baseline Bias	0.259	0.285	0.037	7.756	< .001
	Model 2	Move-In Bias	0.355	1.099	0.106	10.352	< .001
		Baseline Bias	0.254	0.280	0.037	7.601	< .001
		Flow	− 0.005	− 0.001	0.004	− 0.154	.878
		Move-In * Flow	0.095	0.105	0.044	2.352	.019
2011–2015	Model 1	Move-In Bias	0.201	0.688	0.112	6.135	< .001
		Baseline Bias	0.378	0.389	0.034	11.546	< .001
	Model 2	Move-In Bias	0.198	0.677	0.113	6.016	< .001
		Baseline Bias	0.377	0.389	0.034	11.016	< .001
		Flow	− 0.031	− 0.005	0.004	− 1.074	.283
		Move-In * Flow	0.018	0.018	0.054	0.338	.735
2016–2020	Model 1	Move-In Bias	0.520	1.129	0.053	21.300	< .001
		Baseline Bias	0.334	0.344	0.025	13.688	< .001
	Model 2	Move-In Bias	0.519	1.127	0.053	21.346	< .001
		Baseline Bias	0.331	0.340	0.025	13.528	< .001
		Flow	− 0.015	− 0.002	0.002	− 0.673	.501
		Move-In * Flow	0.071	0.073	0.025	2.983	.003

### Migration flow has mixed effects on implicit and explicit bias

#### Implicit bias

For implicit racial bias, there was a significant interaction between migration flow and move-in bias in 2006–2010, β = 0.14, *t*(707) = 2.68, *p* = 0.008. However, only a significant main effect of migration flow was found in 2011–2015 (β = − 0.003, *t*(884) = -2.54 *p* < 0.001) and in 2016–2020 (β = − 0.002, *t*(966) = -2.36, *p* < 0.001). In contrast with our hypothesis, the effect of move-in bias on implicit bias was not consistently moderated by the proportion of incoming movers across three datasets.

#### Explicit bias

For explicit racial bias, there was again a significant interaction between migration flow and move-in bias in 2006–2010 (β = 0.11, *t*(707) = 2.35, *p* = 0.019) and in 2016–2020 (β = 0.07, *t*(966) = 2.98, *p* = 0.003). However, neither the main effect of migration flow (β = − 0.01, *t*(884) = -1.07, *p* = 0.283) nor the interaction with move-in bias (β = 0.02, *t*(884) = 0.34, *p* = 0.735) was significant in 20,111–2015. Similar to implicit bias, the effect of move-in bias was not consistently moderated by the proportion of incoming movers across three datasets. Details of all the results can be found in Model 2 of Tables 2 and 3.

### Robustness check

**Fig. 3:**
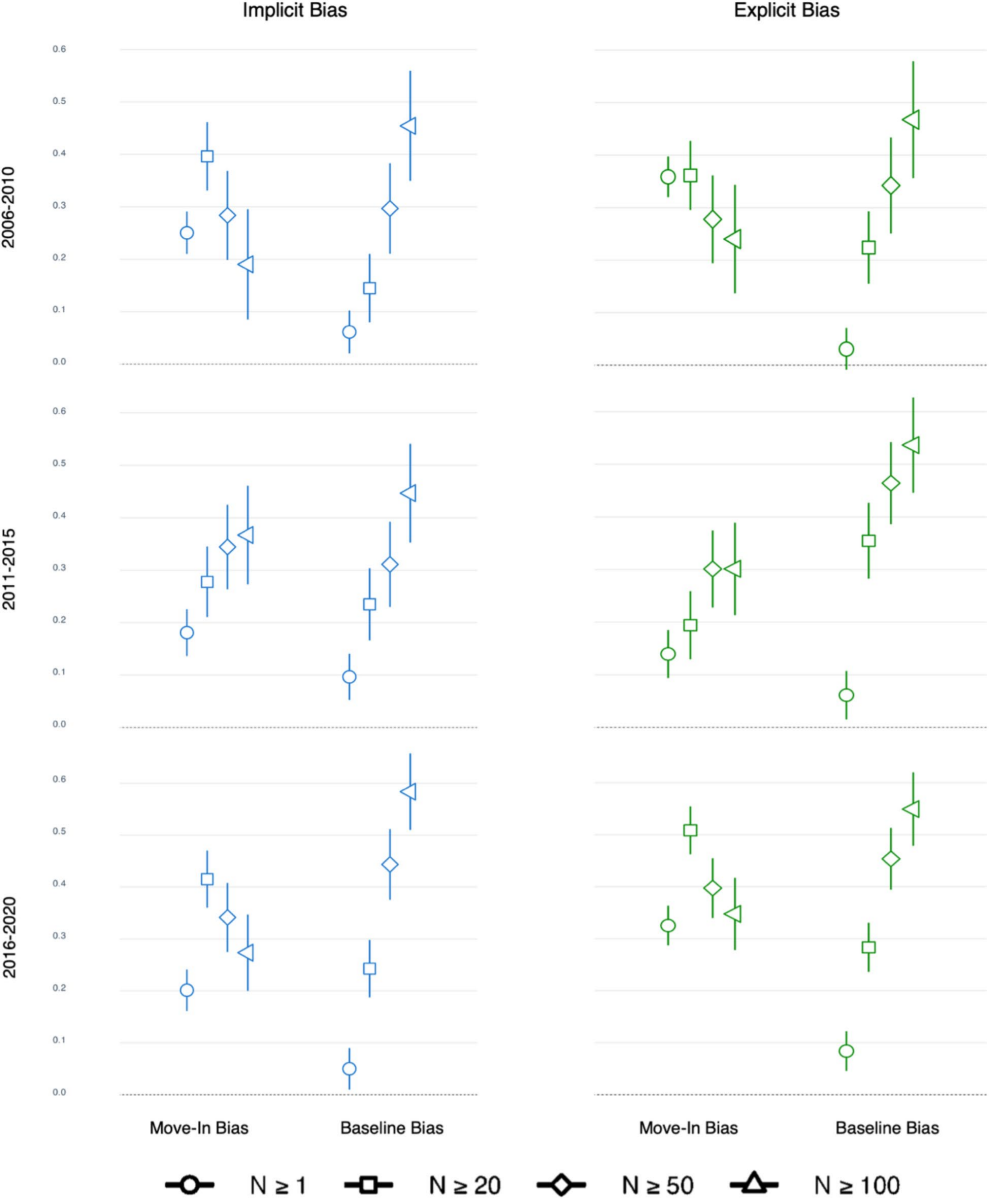
Robustness Check. Standardized regression coefficient (*B*) of move-in bias and baseline bias in Model 1, predicting county-level outcome bias across different inclusion thresholds. Error bar represents 95% confidence internal.

We ran the above analyses (Models 1 and 2) with different inclusion criteria for the minimum number of respondents per county (*N* = 1, 50, 100). Overall, the results are consistent with what we found with a participant threshold of *N* = 20. The effects of move-in bias and baseline bias predicting outcome bias were reliable across all inclusion criteria. The interaction between move-in bias and migration flow was however not significant in most analyses. Figure 3 shows the standardized regression coefficients of move-in bias and baseline bias in predicting outcome bias across different inclusion thresholds. See Table S5a and b in the supplementary material for details.

### A mini Meta-analysis across three time periods

We performed a mini meta-analyzed^43^ with the effect sizes from the model results above using a fixed effects approach. All correlations were weighted by sample size, and transformed to Fisher’s z for normality in analyses, and then converted back to Pearson correlation for presentation. The meta-analytic effect size for move-in bias on outcome bias was significant based on Stouffer’s Z-test (Goh et al., 2016). Aggregated move-in bias was positively correlated with both implicit (Mean *r* = 0.38, *Z* = 20.47, *p* < 0.001 (two tailed), 95% CI [0.35, 0.42]) and explicit outcome bias (Mean *r* = 0.40, *Z* = 21.38, *p* < 0.001 (two tailed), 95% CI [0.37, 0.43]). However, the interaction between move-in bias and migration flow was only significant on explicit outcome bias (Mean *r* = 0.07, *Z* = 3.35, *p* < 0.001 (two tailed), 95% CI [0.03, 0.11]), but not implicit outcome bias (Mean *r* = 0.04, *Z* = 1.77, *p* = 0.077 (two tailed), 95% CI [− 0.00, 0.07]).

## General discussion

In the present research, we examined the impact of people relocating from one county to another in the U.S. on racial attitudes in their new county, with three migration datasets covering the period from 2006 to 2020. Consistent with our predication, move-in bias positively predicts county-level racial bias after migration. This effect was observed even after accounting for existing county-level racial bias. Our findings suggest that new residents bring their racial attitudes from previous counties with them, which then over time influence the overall racial attitudes in their new county. This process happens regardless of what the county’s initial attitudes were like before they moved in.

The robustness of the move-in bias effect—bias brought into a county by people moving into it—was demonstrated across multiple sample inclusion criteria (*N* = 1, 20, 50, 100) and over three distinct time periods (2006–2010, 2011–2015, and 2016–2020). As shown in Fig. 3, all effects are reliably different from zero, although their magnitude varies (see the supplementary material for more detailed discussion and speculations on why these estimates may vary.) As suggested by Charlesworth and Banaji^44^, the U.S. has been experiencing a decrease in racial bias over the past two decades, with a much more significant reduction in explicit than implicit bias. Interestingly, we observed this trend in explicit bias at the county level, as indicated by the county biases shifting downwards in Fig. 2. Despite this overall decline in explicit bias, the move-in effect remained robust across three time periods. While the general level of racial bias may be decreasing, the influence of new residents’ biases on the receiving county’s bias persists. The magnitudes of move-in bias effects were similar for both explicit and implicit biases, suggesting that this mechanism may have impacted both types of biases in comparable ways. The biases newcomers bring with them influence not only overt, consciously held attitudes (explicit bias) but also less conscious automatic associations (implicit bias) among people in a county, at least after a 5-year period.

Contrary to our prediction, the move-in bias effect was not moderated by the migration flow: the effect did not differ across counties as a function of in-migration relative to the total population. This outcome surprised us because county-level migration flow has been shown to be closely tied to socioeconomic factors. For example, more people tend to move to counties with lower unemployment and poverty rates^45^ and less racial segregation^46^. Given that move-in bias remained robust across various county samples, the scale of migration may have a trivial impact on move-in bias effects.

Previous research has indicated that the racial composition of a county can be a significant factor in shaping racial attitudes within that county, as evidenced by disparities in traffic stop rates^25^. As another robustness check suggested by a reviewer, we also investigated whether the relationship between outcome bias and move-in bias can be accounted for by changes in county-level racial composition (i.e., proportion of White and Black residents) and socioeconomic indices (i.e., unemployment rate and personal income). Across a variety of analyses reported in full in the supplement, the move-in bias effect remains significant, indicating that the influence of movers cannot be fully explained by overall shifts in racial composition or socioeconomic trends.

## Limitation

The conclusions we can draw from this project are limited in several ways. First, we estimated the aggregate biases brought by movers based on the aggregate biases of their prior county of residence. This estimation is grounded by the assumption that people carry the culture of their previous regions with them. However, details of this mechanism remain an empirical question for future research to test. The data we relied on in the present research do not provide insight into the specific individual biases of the movers, nor do we have information about the reasons driving them to move to a particular county. Studies on person-environment fit have shown that people moving out might be less representative of their prior county due to lack of fit, and they may be more similar or aspire to the lifestyle of their new place^5,47^. In fact, the small to moderate correlations between move-in bias and baseline bias (see Table S6 in Supplementary material) suggest the selective migration may play a role in the overall migration process. Most movers tend to relocate to geographically-close areas. Census data show a higher percentage of within-state moves compared to interstate moves^48^. People also choose where to move based on factors that better fit their lifestyles and ideological beliefs^49^, leading to reinforced partisan sorting over time^50^. People who migrate over long distances may share similar traits, such as being more educated and risk friendly^51^. These nuances are not captured in the present study.

Second, due to the nature of census data, our analyses were divided into three 5-year periods. Migration is a continuous and dynamic process, and this segmentation does not align with its inherent nature. Using discrete time intervals may obscure the fluidity and complexity of migration patterns. For instance, individuals who move at the beginning or end of these periods might experience different socioeconomic conditions or policy changes compared to those who move mid-period. Moreover, newcomers’ contribution to county biases after migration likely varies depending on their length of residence in the new county. This rigid segmentation of the census data also limits our ability to capture short-term fluctuations and trends within each period, potentially leading to an oversimplified understanding of migration dynamics.

Third, the data we used in the present study have limited generalizability. Project Implicit visitors are a convenience sample which may not be fully representative of the entire county population. However, given the large sample size (over 28 million respondents) and predictive validity to real-world outcomes^30^, along with the representative nature of the census migration data, we have some confidence that the drawback is unlikely to be a significant problem. Nevertheless, future research should exercise caution and test these findings with other attitude datasets to assess their generalizability and further validate our conclusions. Although our results are consistent across different numbers of counties, even with *N* = 1, our analyses still included only two-thirds of U.S. counties. The excluded counties are possibly less populous and may have experienced no migration flow over the observed time periods. Consequently, we cannot determine how the exclusion of these regions might alter the conclusions of this research.

Lastly, given that we relied on data that were collected in waves over time, the present research provides temporal evidence for the effect of move-in bias on county-level bias. Temporal precedence positions us to make limited claims of causality. However, to make stronger claims of causality we would also need to rule out all possible third variables that might explain the relationship between move-in bias and county-level bias (i.e., internal validity). Because we relied only on measured variables and correlational analyses in the present research, we cannot exclude the possibility of third variables and, thus, make only limited claims of causality here.

### Future directions

One promising avenue for future research lies in unpacking the characteristics of the movers, such as their racial components, individual biases and homogeneity. Another approach would be to examine the variability of biases brought by the movers. Specifically, More heterogeneous groups of incoming residents may have a different impact on regional attitude changes. The present study also opens up questions about factors that predict the magnitude of the move-in bias effect and the trajectory of local cultural change over time. At the micro-level, relocating to a new place substantially changes a person’s physical and interpersonal environment. Future research should explore how movers interact with local residents and transmit their previous biases. Such an investigation could use agent-based simulation models that combine regional indicators such as racial segregation indices or population density to simulate and analyze these interactions. These models could provide valuable insights into the mechanisms of bias transmission and the potential long-term impacts on local communities.

## Concluding remarks

The present research investigates county-level racial bias changes in relation to migration. Our findings underscore the idea that migration can play a significant role in the spread and reinforcement of biases across different regions. County-level racial biases are associated with the aggregated biases of migrants estimated based on where they are from. Notably, while our study highlights changes in regional biases, the present research does not speak to the extent to which local residents’ individual attitudes also change following migration. Though we cannot investigate this with the current study, we speculate that the migration-associated county-level bias change is not simply a case of mover biases “showing up” in new locations. Instead, newcomers interact with established residents and become part of the local social fabric, thereby influencing and potentially contributing to the prevailing attitudes and norms^5^. In the long term, both movers and local residents may adapt their attitudes to reflect the shifting regional culture. We look forward to future research exploring these intricate dynamics to better understand how migration influences both regional and individual biases.

## Supplementary Information

Supplementary Information

## Data Availability

The datasets generated and/or analyzed for the current study are available at https://osf.io/sevcq/?view_only=b40683510d3543daa5a6a809f21f8c68.
