# Supplementary material for: County-to-county migration is associated with county-level racial bias in the United States
Source: Sci Rep. 2025 Feb 21;15:6392. doi: 10.1038/s41598-025-88218-7 (PMC11845736; doi:10.1038/s41598-025-88218-7)
Supplement: Supplementary file 1 — Supplementary Information [file 41598_2025_88218_MOESM1_ESM.docx]

**Supplementary Material for Jin, Calanchini, & Ratliff (2024)**

***Summary***

This document includes six exploratory analyses, 1-4 are pre-registered (for details, see https://osf.io/sevcq/?view_only=b40683510d3543daa5a6a809f21f8c68).

1. Re-run the regression model (Model 2) in the main analyses by adding migration counterflow as a control variable. i.e., Model 3: Outcome bias = Aggregated move-in bias * Migration flow + Baseline bias + Counterflow.
2. Summarize Moran’s *I* test for global spatial dependence in the two models in the main analyses.
3. Examine whether migration flow is positively correlated with county-level bias variability.
4. Re-run the two regression models in the main text (Model 1 and 2) by employing multiple data inclusion criteria (*N* = 0, 50, 100).
5. Correlation matrix of all predictor variables and detect multicollinearity using variance inflation factors.
6. Conduct additional analyses to interpret the varying effect sizes of two predictors shown in Figure 3 of the main text.
7. Re-run the regression model (Model 2) by adding county-level changes in racial composition and socioeconomic indices as covariates.

***Exploratory Analyses***

**1. Counterflow as a Control Variable**

The model results are largely consistent with the main analyses, after adding counterflow as a control variable. Results are summarized in Table S1a and S1b.

The effect of aggregated move-in bias remains significant in all three time periods. For implicit outcome bias, in 2006-2010, β = 1.36, *t*(706) = 11.78, *p* <.001; in 2011-2015, β = 0.75, *t*(883) = 8.45, *p* <.001; and in 2016-2010, β = 0.98, *t*(965) = 14.57, *p* <.001. For explicit outcome bias, in 2006-2010, β = 1.09, *t*(706) = 10.36, *p* <.001; in 2011-2015, β = 0.68, *t*(883) = 6.02, *p* <.001; and in 2016-2020, β = 1.13, *t*(965) = 21.33, *p* <.001. The interaction between aggregated move-in bias and migration flow for implicit outcome bias was again only significant in in 2006-2010, β = 0.13, *t*(706) = 2.48, *p* =.013, but not in 2011-2015, β = -0.05, *t*(883) = -1.09, *p* =.277; or in 2016-2010, β = -0.03, *t*(965) = -0.95, *p* =.344. For explicit outcome bias, consistent with the results in main analyses, the interaction was significant in 2006-2010, β = 0.10, *t*(706) = 2.27, *p* =.021; in 2016-2020, β = 0.07, *t*(965) = 2.78, *p* =.006. But it was not significant in 2011-2015, β = 0.02, *t*(883) = 0.40, *p* =.690.

There were mixed results on the effect of counterflow on outcome racial bias. It was negatively correlated with the implicit outcome bias in 2006-2010 (β = -0.01, *t*(706) = -3.51, *p* <.001) and 2016-2020 (β = -0.00, *t*(965) = -2.66, *p* =.008), but no significant correlation was found in 2011-2015 (β = -0.00 *t*(883) = -0.90, *p* =.371). It was also negatively correlated with the explicit outcome bias in 2006-2010 (β = -0.01, *t*(706) = -2.32, *p* =.024). However, no significant correlation was found in 2011-2015 (β = 0.00 *t*(883) = 0.34, *p* =.737) or 2016-2020 (β = -0.00, *t*(965) = -0.93, *p* =.355).

| **Table S1a. Model Results for Implicit Racial Bias** | | | | | | | |
| --- | --- | --- | --- | --- | --- | --- | --- |
|  |  | **Predictor** | ***B*** | **β** | ***SE*** | ***t*** | ***p*** |
| 2006-2010 | Model 3 | Move-In Bias | 0.401 | 1.363 | 0.116 | 11.777 | <.001 |
|  |  | Baseline Bias | 0.155 | 0.174 | 0.038 | 4.611 | <.001 |
|  |  | Flow | 0.100 | 0.004 | 0.001 | 2.467 | .014 |
|  |  | Move-In * Flow | 0.113 | 0.127 | 0.051 | 2.482 | .013 |
|  |  | Counterflow | -0.096 | -0.007 | 0.002 | -3.513 | <.001 |
|  |  |  |  |  |  |  |  |
| 2011-2015 | Model 3 | Move-In Bias | 0.284 | 0.754 | 0.089 | 8.450 | <.001 |
|  |  | Baseline Bias | 0.256 | 0.208 | 0.027 | 7.671 | <.001 |
|  |  | Flow | -0.048 | -0.002 | 0.002 | -1.137 | 0.256 |
|  |  | Move-In * Flow | -0.060 | -0.048 | 0.045 | -1.087 | 0.277 |
|  |  | Counterflow | -0.039 | -0.002 | 0.002 | -0.895 | 0.371 |
|  |  |  |  |  |  |  |  |
| 2016-2020 | Model 3 | Move-In Bias | 0.413 | 0.980 | 0.067 | 14.570 | <.001 |
|  |  | Baseline Bias | 0.255 | 0.214 | 0.024 | 9.019 | <.001 |
|  |  | Flow | -0.006 | -0.000 | 0.001 | -0.163 | .871 |
|  |  | Move-In * Flow | -0.038 | -0.032 | 0.033 | -0.947 | .344 |
|  |  | Counterflow | -0.092 | -0.004 | 0.001 | -2.656 | .008 |

| **Table S1b. Model Results for Explicit Racial Bias** | | | | | | | |  |
| --- | --- | --- | --- | --- | --- | --- | --- | --- |
|  |  | **Predictor** | ***B*** | **β** | ***SE*** | ***t*** | ***p*** | |
| 2006-2010 | Model 3 | Move-In Bias | 0.351 | 1.087 | 0.106 | 10.258 | <.001 | |
|  |  | Baseline Bias | 0.239 | 0.263 | 0.037 | 7.055 | <.001 | |
|  |  | Flow | 0.051 | 0.006 | 0.005 | 1.253 | .211 | |
|  |  | Move-In * Flow | 0.091 | 0.100 | 0.044 | 2.266 | .021 | |
|  |  | Counterflow | -0.096 | -0.014 | 0.006 | -2.316 | .024 | |
|  |  |  |  |  |  |  |  | |
| 2011-2015 | Model 3 | Move-In Bias | 0.198 | 0.679 | 0.113 | 6.022 | <.001 | |
|  |  | Baseline Bias | 0.378 | 0.389 | 0.034 | 11.518 | <.001 | |
|  |  | Flow | -0.041 | -0.006 | 0.006 | -0.989 | 0.323 | |
|  |  | Move-In * Flow | 0.021 | 0.022 | 0.055 | 0.398 | 0.690 | |
|  |  | Counterflow | 0.014 | 0.002 | 0.007 | 0.336 | 0.737 | |
|  |  |  |  |  |  |  |  | |
| 2016-2020 | Model 3 | Move-In Bias | 0.518 | 1.126 | 0.053 | 21.329 | <.001 | |
|  |  | Baseline Bias | 0.330 | 0.339 | 0.025 | 13.483 | <.001 | |
|  |  | Flow | 0.002 | 0.000 | 0.003 | 0.063 | .950 | |
|  |  | Move-In * Flow | 0.067 | 0.069 | 0.025 | 2.776 | .006 | |
|  |  | Counterflow | -0.027 | -0.004 | 0.004 | -0.925 | .355 | |

**2. Testing for Spatial Dependence**

Data aggregated at county level may be correlated with each other via spatial proximity, thus violating the assumption of independence in regression. Therefore, we must examine the spatial autocorrelation in our modelling. We first created a spatial weights matrix based on the *k*-nearest neighbor of each county (*k* = 4). We chose distance-based matrix due to the dispersed distribution of counties in our data (*N* = 712, 889, 971 for three time periods respectively, which is less than 1/3 of the total counties in US). The longitude-latitude decimal degrees of county centroid were used as point coordinates. We then ran the Moran’s *I* test on the models in the main analyses and found no significant results, suggesting no spatial autocorrelation was found in the model residuals. Results are summarized in Table S2 below.

| **Table S2. Moran's *I* Test for Global Spatial Autocorrelation** | | | | |
| --- | --- | --- | --- | --- |
|  |  |  | **Observed *I*** | ***p*** |
| **Implicit Racial Bias** | 2006-2010 | Model 1 | -0.119 | .547 |
|  |  | Model 2 | -0.093 | .537 |
|  |  | Model 3 | -0.053 | .521 |
|  |  |  |  |  |
|  | 2011-2015 | Model 1 | -0.061 | .524 |
|  |  | Model 2 | -0.311 | .622 |
|  |  | Model 3 | -0.324 | .627 |
|  |  |  |  |  |
|  | 2016-2020 | Model 1 | 0.850 | .198 |
|  |  | Model 2 | 0.844 | .200 |
|  |  | Model 3 | 0.937 | .174 |
|  |  |  |  |  |
| **Explicit Racial Bias** | 2006-2010 | Model 1 | 1.023 | .153 |
|  |  | Model 2 | 1.067 | .143 |
|  |  | Model 3 | 1.110 | .134 |
|  |  |  |  |  |
|  | 2011-2015 | Model 1 | -0.424 | .664 |
|  |  | Model 2 | -0.491 | .688 |
|  |  | Model 3 | -0.475 | .683 |
|  |  |  |  |  |
|  | 2016-2020 | Model 1 | -0.719 | .764 |
|  |  | Model 2 | -0.759 | .776 |
|  |  | Model 3 | -0.740 | .770 |

**3. Mixed Findings on the Correlation between Migration Flow and County-Level Bias Variability**

We ran a series of simple regression models to test whether counties with higher migration flow have higher implicit and explicit racial bias variability. Bias variability is defined as the standard deviation of the bias averaged across 5 years. The results are mixed on whether there is a significant correlation between the two variables across three time periods. For implicit bias variability, in 2006-2010, the migration flow is not significantly correlated with bias variability, β = 0.00, *t*(2100) = 1.15, *p* =.250. However, there is a positive correlation between them in 2011-2015, β = 0.00, *t*(1825) = 2.03, *p* =.043, and in 2016-2020, β = 0.00, *t*(2346) = 3.43, *p* <.001. For explicit bias variability, in 2006-2010, again no correlation was found, β = 0.00, *t*(2100) = 1.34, *p* =.181, or in 2016-2020, β = 0.00, *t*(2346) = 0.74, *p* =.460. However, a negative correlation was found in 2011-2015, β = -0.00, *t*(1825) = -2.21, *p* =.027. Despite the statistical significance of some associations, it is important to note the small standardized coefficients (B) in every model. This suggests that the observed effects are likely trivial in nature.

| **Table S3. Model Results for Racial Bias Variability** | | | | | | | | |
| --- | --- | --- | --- | --- | --- | --- | --- | --- |
|  |  |  | **Predictor** | ***B*** | **β** | ***SE*** | ***t*** | ***p*** |
| **Implicit Bias**  **Variability** | 2006-2010 | Model 4 | Flow | 0.025 | 0.000 | 0.000 | 1.150 | .250 |
|  | 2011-2015 | Model 4 | Flow | 0.047 | 0.001 | 0.000 | 2.027 | .043 |
|  | 2016-2020 | Model 4 | Flow | 0.071 | 0.001 | 0.000 | 3.425 | <.001 |
|  |  |  |  |  |  |  |  |  |
| **Explicit Bias**  **Variability** | 2006-2010 | Model 4 | Flow | 0.029 | 0.003 | 0.002 | 1.339 | .181 |
|  | 2011-2015 | Model 4 | Flow | -0.052 | -0.005 | 0.002 | -2.207 | .027 |
|  | 2016-2020 | Model 4 | Flow | 0.015 | 0.001 | 0.002 | 0.739 | .460 |

**4. Results of Main Analyses with Multiple Inclusion Criteria**

In the main text, we conducted all analyses on counties with a minimum of *N* = 20 respondents from the Project Implicit data. Limiting the number of respondents substantially decreased the number of counties included in the analyses (see Table S4), which could have reduced statistical power. Therefore, we explored the two main hypotheses using multiple *N* thresholds to assess the consistency of results across different selection criteria.

All model results support our hypothesis that the aggregated move-in bias and baseline bias positively predict the implicit and explicit outcome racial bias (except for 2006-2010, N=1 of baseline bias). For implicit bias, move-in bias *B* = 0.18 – 0.43, baseline bias *B* = 0.05 – 0.59. For explicit bias, move-in bias *B* = 0.15 – 0.52, baseline bias *B* = 0.03 – 0.57. Although there are consistent positive effects of move-in bias and baseline bias, the magnitude of the effect ranges from small to medium. Interestingly, the magnitude of baseline bias effect increases as the number of respondents increases, suggesting it has a stronger correlation with the outcome bias in counties with larger *Ns*. This pattern of results is consistent with the findings in Stelter et al. (2022). No such relationship was observed in the magnitude of move-in bias.

Similar to the main text, the overall results showed a mixed pattern of results on the interaction between move-in bias and migration flow. For implicit bias, interaction *B* = -0.14 – 0.17; for explicit bias, interaction *B* = -0.08 – 0.13. Thus, we did not find support for the interaction effect that the effect of move-in bias depends on how many people have moved in.

| **Table S4. Descriptive Statistics** | | | |
| --- | --- | --- | --- |
| **Inclusion Criteria** | ***N* _2006-2010_** | ***N* _2011-2015_** | ***N* _2016-2020_** |
| ≥ 1 respondent | 2254 | 2116 | 2354 |
| ≥ 20 respondents | 712 | 889 | 971 |
| ≥ 50 respondents | 424 | 544 | 575 |
| ≥ 100 respondents | 273 | 349 | 383 |

| **Table S5a. Model Results with Different *N* for Implicit Bias** | | | | | | |
| --- | --- | --- | --- | --- | --- | --- |
|  |  | **Predictor** | ***B_1_*** | ***B_20_*** | ***B_50_*** | ***B_100_*** |
| 2006-2010 | Model 1 | Move-In Bias | 0.241*** | 0.425*** | 0.306*** | 0..216*** |
|  |  | Baseline Bias | 0.069*** | 0.173*** | 0.341*** | 0.479*** |
|  |  |  |  |  |  |  |
|  | Model 2 | Move-In Bias | 0.242*** | 0.411*** | 0.284*** | 0.221*** |
|  |  | Baseline Bias | 0.069*** | 0.176*** | 0.339*** | 0.478*** |
|  |  | Flow | 0.004 | 0.016 | 0.037 | 0.058 |
|  |  | Move-In * Flow | 0.015 | 0.123** | 0.171** | 0.096 |
|  |  |  |  |  |  |  |
| 2011-2015 | Model 1 | Move-In Bias | 0.176*** | 0.293*** | 0.359*** | 0.349*** |
|  |  | Baseline Bias | 0.053*** | 0.255*** | 0.327*** | 0.429*** |
|  |  |  |  |  |  |  |
|  | Model 2 | Move-In Bias | 0.176*** | 0.286*** | 0.350*** | 0.330*** |
|  |  | Baseline Bias | 0.055*** | 0.256*** | 0.328*** | 0.444*** |
|  |  | Flow | -0.044* | -0.076* | -0.080* | -0.133** |
|  |  | Move-In * Flow | 0.020 | -0.051 | -0.028 | -0.141* |
|  |  |  |  |  |  |  |
| 2016-2020 | Model 1 | Move-In Bias | 0.215*** | 0.425*** | 0.342*** | 0.268*** |
|  |  | Baseline Bias | 0.067*** | 0.261*** | 0.463*** | 0.593*** |
|  |  |  |  |  |  |  |
|  | Model 2 | Move-In Bias | 0.209*** | 0.419*** | 0.341*** | 0.268*** |
|  |  | Baseline Bias | 0.060*** | 0.255*** | 0.462*** | 0.594*** |
|  |  | Flow | 0.003 | -0.063* | -0.050 | -0.034 |
|  |  | Move-In * Flow | -0.073 | -0.017 | -0.034 | -0.051 |

*p* < 0.001 ***, *p* < 0.01 **, *p* < 0.05 *

| **Table S5b. Model Results with Different *N* for Explicit Bias** | | | | | | |
| --- | --- | --- | --- | --- | --- | --- |
|  |  | **Predictor** | ***B_1_*** | ***B_20_*** | ***B_50_*** | ***B_100_*** |
| 2006-2010 | Model 1 | Move-In Bias | 0.358*** | 0.375*** | 0.305*** | 0.249*** |
|  |  | Baseline Bias | 0.027 | 0.259*** | 0.408*** | 0.486*** |
|  |  |  |  |  |  |  |
|  | Model 2 | Move-In Bias | 0.359*** | 0.355*** | 0.284*** | 0.252*** |
|  |  | Baseline Bias | 0.028*** | 0.254*** | 0.401*** | 0.497*** |
|  |  | Flow | -0.001 | -0.005 | 0.065 | 0.135** |
|  |  | Move-In * Flow | 0.037 | 0.095* | 0.127** | 0.132* |
|  |  |  |  |  |  |  |
| 2011-2015 | Model 1 | Move-In Bias | 0.153*** | 0.201*** | 0.303*** | 0.297*** |
|  |  | Baseline Bias | 0.077*** | 0.378*** | 0.469*** | 0.530*** |
|  |  |  |  |  |  |  |
|  | Model 2 | Move-In Bias | 0.150*** | 0.198*** | 0.299*** | 0.302*** |
|  |  | Baseline Bias | 0.077*** | 0.377*** | 0.466*** | 0.540*** |
|  |  | Flow | -0.009 | -0.031 | -0.009 | 0.005 |
|  |  | Move-In * Flow | -0.023 | 0.018 | 0.031 | -0.077 |
|  |  |  |  |  |  |  |
| 2016-2020 | Model 1 | Move-In Bias | 0.334*** | 0.520*** | 0.396*** | 0.339*** |
|  |  | Baseline Bias | 0.095*** | 0.334*** | 0.501*** | 0.573*** |
|  |  |  |  |  |  |  |
|  | Model 2 | Move-In Bias | 0.335*** | 0.519*** | 0.393*** | 0.336*** |
|  |  | Baseline Bias | 0.095*** | 0.331*** | 0.498*** | 0.575*** |
|  |  | Flow | 0.012 | -0.015 | -0.014 | 0.054 |
|  |  | Move-In * Flow | 0.012 | 0.071** | -0.045 | -0.012 |

*p* < 0.001 ***, *p* < 0.01 **, *p* < 0.05 *

**5. Correlation matrix of Predictors and Multicollinearity Diagnosis**

Multicollinearity is a potential problem in all regression analyses. Here we present the correlation matrix of predictors in our main analyses, and check for multicollinearity following the advice from Thompson and colleagues^1^.

We found weak to moderate correlations between move-in bias and baseline bias for both implicit and explicit bias in all three datasets; weak correlation between move-in bias and migration flow in the majority of results; and lastly, inconsistent results on the correlation between baseline bias and migration flow. See Table S6 for details. We thus centered all the variables before running the regression analyses. We then check for multicollinearity of the predictors in our model with variance inflation factors (VIF). The results are presented in Table S7a and S7b. None of the models have a VIF larger than 2, indicating that multicollinearity is unlikely a problem in our models.

**Reference**

1. Thompson, C. G., Kim, R. S., Aloe, A. M. & Becker, B. J. Extracting the Variance Inflation Factor and Other Multicollinearity Diagnostics from Typical Regression Results. *Basic Appl. Soc. Psychol.* **39**, 81–90 (2017).

| **Table S6. Correlation Matrix of Predictor Variables** | | | | | |
| --- | --- | --- | --- | --- | --- |
|  |  |  | 1 | 2 | 3 |
| **Implicit Bias** | 2006-2010 | 1. Move-in Bias | — |  |  |
|  |  | 2. Baseline Bias | 0.236*** | — |  |
|  |  | 3. Flow | -0.150*** | -0.080* | — |
|  |  |  |  |  |  |
|  | 2011-2015 | 1. Move-in Bias | — |  |  |
|  |  | 2. Baseline Bias | 0.462*** | — |  |
|  |  | 3. Flow | -0.103** | -0.039 | — |
|  |  |  |  |  |  |
|  | 2016-2020 | 1. Move-in Bias | — |  |  |
|  |  | 2. Baseline Bias | 0.365*** | — |  |
|  |  | 3. Flow | -0.124** | -0.133*** | — |
|  |  |  |  |  |  |
| **Explicit Bias** | 2006-2010 | 1. Move-in Bias | — |  |  |
|  |  | 2. Baseline Bias | 0.232*** | — |  |
|  |  | 3. Flow | -0.120* | -0.055 | — |
|  |  |  |  |  |  |
|  | 2011-2015 | 1. Move-in Bias | — |  |  |
|  |  | 2. Baseline Bias | 0.459*** | — |  |
|  |  | 3. Flow | -0.073* | -0.030 | — |
|  |  |  |  |  |  |
|  | 2016-2020 | 1. Move-in Bias | — |  |  |
|  |  | 2. Baseline Bias | 0.432*** | — |  |
|  |  | 3. Flow | -0.054 | -0.118*** | — |

*p* < 0.001 ***, *p* < 0.01 **, *p* < 0.05 *

| **Table S7a. Multicollinearity Diagnosis for Implicit bias** | | | | |
| --- | --- | --- | --- | --- |
|  |  | **Predictor** | **Tolerance** | **VIF** |
| 2006-2010 | Model 1 | Move-In Bias | 0.944 | 1.059 |
|  |  | Baseline Bias | 0.944 | 1.059 |
|  |  |  |  |  |
|  | Model 2 | Move-In Bias | 0.901 | 1.110 |
|  |  | Baseline Bias | 0.942 | 1.062 |
|  |  | Flow | 0.965 | 1.036 |
|  |  | Move-In * Flow | 0.956 | 1.046 |
|  |  |  |  |  |
| 2011-2015 | Model 1 | Move-In Bias | 0.787 | 1.271 |
|  |  | Baseline Bias | 0.787 | 1.271 |
|  |  |  |  |  |
|  | Model 2 | Move-In Bias | 0.777 | 1.287 |
|  |  | Baseline Bias | 0.786 | 1.272 |
|  |  | Flow | 0.989 | 1.011 |
|  |  | Move-In * Flow | 0.993 | 1.007 |
|  |  |  |  |  |
| 2016-2020 | Model 1 | Move-In Bias | 0.867 | 1.153 |
|  |  | Baseline Bias | 0.867 | 1.153 |
|  |  |  |  |  |
|  | Model 2 | Move-In Bias | 0.860 | 1.163 |
|  |  | Baseline Bias | 0.858 | 1.166 |
|  |  | Flow | 0.974 | 1.027 |
|  |  | Move-In * Flow | 0.995 | 1.005 |

| **Table S7b. Results of Multicollinearity Diagnosis** | | | | |
| --- | --- | --- | --- | --- |
|  |  | **Predictor** | **Tolerance** | **VIF** |
| 2006-2010 | Model 1 | Move-In Bias | 0.946 | 1.057 |
|  |  | Baseline Bias | 0.946 | 1.057 |
|  |  |  |  |  |
|  | Model 2 | Move-In Bias | 0.890 | 1.124 |
|  |  | Baseline Bias | 0.942 | 1.062 |
|  |  | Flow | 0.969 | 1.032 |
|  |  | Move-In * Flow | 0.919 | 1.089 |
|  |  |  |  |  |
| 2011-2015 | Model 1 | Move-In Bias | 0.789 | 1.268 |
|  |  | Baseline Bias | 0.789 | 1.268 |
|  |  |  |  |  |
|  | Model 2 | Move-In Bias | 0.782 | 1.278 |
|  |  | Baseline Bias | 0.787 | 1.270 |
|  |  | Flow | 0.995 | 1.005 |
|  |  | Move-In * Flow | 0.990 | 1.011 |
|  |  |  |  |  |
| 2016-2020 | Model 1 | Move-In Bias | 0.813 | 1.230 |
|  |  | Baseline Bias | 0.813 | 1.230 |
|  |  |  |  |  |
|  | Model 2 | Move-In Bias | 0.813 | 1.244 |
|  |  | Baseline Bias | 0.804 | 1.230 |
|  |  | Flow | 0.983 | 1.018 |
|  |  | Move-In * Flow | 0.995 | 1.005 |

**6. Interpretation on the varying effect sizes in Figure 3**

Figure 3 in the main text shows that both move-in bias and baseline bias effects are significantly different from zero across sample sizes (*N* = 1, 20, 50,100). However, the magnitudes of effects varied across criteria and merit some discussion. The effect sizes of baseline bias increase as *N* increases whereas the effect sizes of move-in bias, though always significant, were inconsistent and somewhat decreasing in 2 out of 3 datasets.

First, the inclusion criteria applied leads to an unavoidable trade-off between sample size and bias estimation accuracy. Sample size (number of counties) becomes smaller with increasing response thresholds (see Table S4), which in turn should make the regression results less reliable. On the other hand, counties with more respondents offer more stable and accurate bias estimates, which may explain the increasing effect sizes for baseline bias with higher *N*s. However, analysis on counties with more respondents could just be subgroup analysis on counties with larger populations (e.g., urban areas) or counties with more people registered for Project Implicit (e.g., where universities are located). The effect size of move-in bias might be unstable due to the characteristics of the included counties. For the present study, these two factors might both contribute to the varying effect sizes that we observed, and we cannot tease them apart.

Second, though we pre-registered baseline bias as a control variable, the overall changing pattern of two predictors in their effect sizes may suggest an interaction effect. Therefore, we run the regression analyses with an interaction effect (model: Outcome bias = Aggregated move-in bias * Migration flow) for all *N*s. The majority of the results somewhat support the interaction (see Table S8), such that counties with higher baseline bias show smaller effect of move-in bias. This pattern of results suggests that counties that are more biased before migration are less susceptible to mover brought biases. Figure S1, depicting the interaction effect, is included below. This interpretation should be treated with caution because the interaction effect was not pre-registered, and the results across *N*s and timeframes are less consistent.

**Table S8. Interaction between Move-in Bias and Baseline Bias**

|  |  | ***B_1_*** | ***B_20_*** | ***B_50_*** | ***B_100_*** |
| --- | --- | --- | --- | --- | --- |
| 2006-2010 | Implicit | -0.020* | -0.164*** | -0.145*** | -0.098* |
|  | Explicit | 0.007 | -0.079** | -0.084** | -0.031 |
| 2011-2015 | Implicit | -0.020 | -0.039 | -0.028 | 0.033 |
|  | Explicit | -0.034* | -0.029 | -0.006 | 0.009 |
| 2016-2020 | Implicit | -0.075*** | -0.102*** | -0.059** | -0.085** |
|  | Explicit | -0.078*** | -0.146*** | -0.117*** | -0.082*** |


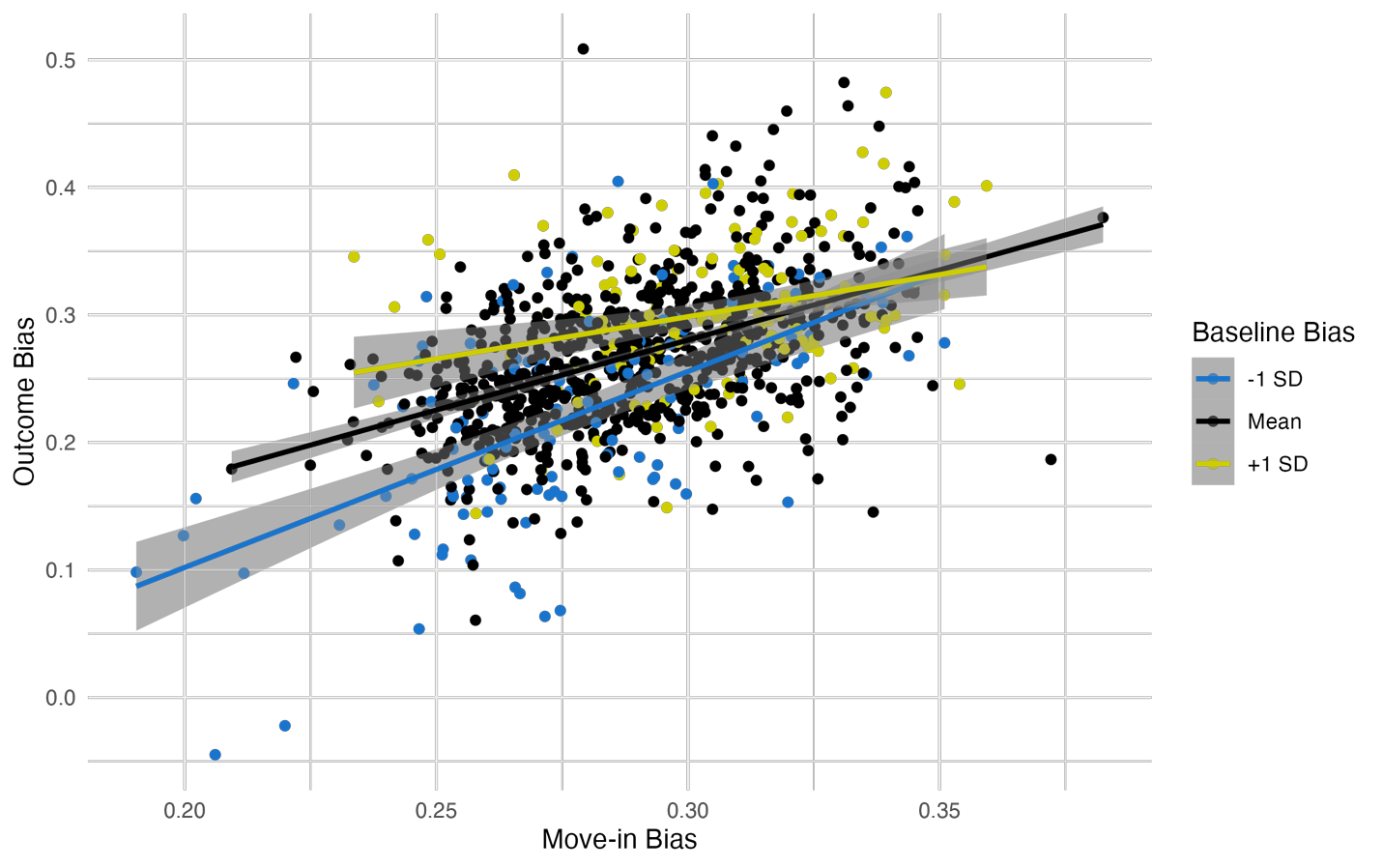


**Figure S1. *Interaction between Move-in Bias and Baseline Bias in Predicting Outcome Bias*.** This figure was generated with implicit racial bias in 2016-2020 and *N*=20. All the other interactions in Table S8 follow similar pattern. Counties with lower baseline bias show stronger effect of move-in bias in predicting outcome bias after migration.

**7. Add County-Level Racial Composition and Socioeconomic Indices As Covariates**

As another robustness check suggested by a reviewer, we also investigated whether the relationship between outcome bias and move-in bias can be accounted for by changes in county-level racial composition (i.e., proportion of White and Black residents) and socioeconomic indices (i.e., unemployment rate and personal income).

**County-level racial composition changes (Black vs. White)**

Model 4: Outcome bias = Aggregated move-in bias + Baseline bias + %ChangeWhite + %ChangeBlack

We ran a model 4 to control for the demographic changes in White (non-Hispanic), and Black population in a county. Changes were calculated using the percentage of population the year after the 5-year period minus the percentage in the year before the period. For example, to examine changes in the White population from 2016 to 2020, we calculated the difference between the percentage of the White population in 2021 and 2015 within each county. Importantly, we do not have strong predictions about whether and how changes in racial demographics might relate to outcome bias. That said, Ekstrom et al., (2022) suggests that county-level racial bias may be positively related to the proportion of White residents but negatively related to the proportion of Black residents. Instead, the purpose of running these additional models was to observe whether the relationship between aggregated move-in bias and outcome bias persists after controlling for changes in racial demographics. And that’s exactly what we found: aggregated move-in bias remains significantly related to outcome bias in all models. Table S9 summarizes findings for implicit bias, and S10 for explicit anti-Black bias.

**County-level socioeconomic changes**

We included both negative economic index (Unemployment Rate) and positive index (Personal Income)^[[1]](#footnote-2)^ in the following models. Five-year average unemployment rate or personal income (m_Unemployment and m_Income) are measuring the overall economic climate, while 5-year changes in unemployment rate or personal income (c_Unemployment and c_Income) are measuring shifts in economic stability.

Model 5: Outcome bias = Aggregated move-in bias + Baseline bias + m_Unemployment

Model 6: Outcome bias = Aggregated move-in bias + Baseline bias + c_Unemployment

Model 7: Outcome bias = Aggregated move-in bias + Baseline bias + m_Income

Model 8: Outcome bias = Aggregated move-in bias + Baseline bias + c_Income

Model results are summarized in Table S11 and S12. We cannot confidently identify a clear or consistent pattern of relationships between economic indices and anti-Black bias; however, the aggregated move-in bias remains significantly related in all models.

In summary, in response to the reviewer’s comment, we ran models controlling for county-level racial composition, unemployment rate, and personal income. Consistent with our previous findings, we found that the move-in bias effect reported in the manuscript remained robust, significantly correlating with county-level racial bias across all the models we ran. To fully disentangle the mechanisms underlying the move-in bias effect, future studies would benefit from new data focusing on the characteristics of migrants instead of county-level covariates.

**Table S9 Model Results for Racial Composition Changes and Implicit Bias**

|  |  | **Predictor** | ***B*** | **β** | ***SE*** | ***t*** | ***p*** |
| --- | --- | --- | --- | --- | --- | --- | --- |
| 2006-2010 | Model 4 | Move-In Bias | 0.248 | 1.003 | 0.222 | 4.513 | <.001 |
|  |  | Baseline Bias | 0.318 | 0.376 | 0.065 | 5.776 | <.001 |
|  |  | %ChangeWhite | -0.080 | -0.004 | 0.003 | -1.259 | .209 |
|  |  | %ChangeBlack | -0.145 | -0.009 | 0.004 | -2.272 | .024* |
|  |  |  |  |  |  |  |  |
| 2011-2015 | Model 4 | Move-In Bias | 0.267 | 0.709 | 0.092 | 7.706 | <.001 |
|  |  | Baseline Bias | 0.258 | 0.210 | 0.027 | 7.744 | <.001 |
|  |  | %ChangeWhite | 0.106 | 0.006 | 0.002 | 2.980 | .003** |
|  |  | %ChangeBlack | 0.039 | 0.003 | 0.003 | 1.128 | .259 |
|  |  |  |  |  |  |  |  |
| 2016-2020 | Model 4 | Move-In Bias | 0.422 | 1.001 | 0.072 | 13.971 | <.001 |
|  |  | Baseline Bias | 0.262 | 0.219 | 0.024 | 9.268 | <.001 |
|  |  | %ChangeWhite | -0.013 | 0.001 | 0.001 | -0.359 | .720 |
|  |  | %ChangeBlack | -0.067 | -0.004 | 0.002 | -2.030 | .043* |

**Table S10 Model Results for Racial Composition Changes and Explicit Bias**

|  |  | **Predictor** | ***B*** | **β** | ***SE*** | ***t*** | ***p*** |
| --- | --- | --- | --- | --- | --- | --- | --- |
| 2006-2010 | Model 4 | Move-In Bias | 0.250 | 0.828 | 0.165 | 5.010 | <.001 |
|  |  | Baseline Bias | 0.486 | 0.504 | 0.052 | 9.763 | <.001 |
|  |  | %ChangeWhite | 0.002 | 0.000 | 0.007 | 0.034 | .973 |
|  |  | %ChangeBlack | 0.010 | 0.002 | 0.011 | 0.179 | .858 |
|  |  |  |  |  |  |  |  |
| 2011-2015 | Model 4 | Move-In Bias | 0.148 | 0.508 | 0.117 | 4.346 | <.001 |
|  |  | Baseline Bias | 0.389 | 0.401 | 0.033 | 11.982 | <.001 |
|  |  | %ChangeWhite | 0.142 | 0.030 | 0.007 | 4.059 | <.001*** |
|  |  | %ChangeBlack | -0.008 | -0.002 | 0.011 | -0.224 | .823 |
|  |  |  |  |  |  |  |  |
| 2016-2020 | Model 4 | Move-In Bias | 0.519 | 1.127 | 0.055 | 20.569 | <.001 |
|  |  | Baseline Bias | 0.331 | 0.340 | 0.025 | 13.625 | <.001 |
|  |  | %ChangeWhite | -0.022 | -0.003 | 0.004 | -0.755 | .045 |
|  |  | %ChangeBlack | -0.098 | -0.020 | 0.006 | -3.589 | <.001*** |

**Table S11 Model Results for Socioeconomic Changes and Implicit Bias**

|  |  | **Predictor** | ***B*** | **β** | ***SE*** | ***t*** | ***p*** |
| --- | --- | --- | --- | --- | --- | --- | --- |
| 2006-2010 | Model 5 | Move-In Bias | 0.418 | 1.417 | 0.114 | 12.457 | <.001 |
|  |  | Baseline Bias | 0.171 | 0.191 | 0.037 | 5.103 | <.001 |
|  |  | M_Unemploy | -0.099 | -0.004 | 0.001 | -3.048 | .002** |
|  |  |  |  |  |  |  |  |
|  | Model 6 | Move-In Bias | 0.426 | 1.446 | 0.114 | 12.598 | <.001 |
|  |  | Baseline Bias | 0.174 | 0.195 | 0.038 | 5.164 | <.001 |
|  |  | C_Unemploy | 0.022 | 0.001 | 0.001 | 0.679 | .498 |
|  |  |  |  |  |  |  |  |
|  | Model 7 | Move-In Bias | 0.467 | 1.524 | 0.114 | 13.324 | <.001 |
|  |  | Baseline Bias | 0.176 | 0.195 | 0.039 | 5.052 | <.001 |
|  |  | M_Income | 0.126 | 0.001 | 0.000 | 3.711 | <.001*** |
|  |  |  |  |  |  |  |  |
|  | Model 8 | Move-In Bias | 0.448 | 1.461 | 0.115 | 12.700 | <.001 |
|  |  | Baseline Bias | 0.183 | 0.203 | 0.039 | 5.223 | <.001 |
|  |  | C_Income | 0.059 | 0.001 | 0.001 | 1.737 | 0.083 |
|  |  |  |  |  |  |  |  |
| 2011-2015 | Model 5 | Move-In Bias | 0.290 | 0.768 | 0.089 | 8.620 | <.001 |
|  |  | Baseline Bias | 0.249 | 0.202 | 0.028 | 7.325 | <.001 |
|  |  | M_Unemploy | -0.032 | -0.001 | 0.001 | -1.052 | .293 |
|  |  |  |  |  |  |  |  |
|  | Model 6 | Move-In Bias | 0.292 | 0.775 | 0.089 | 8.739 | <.001 |
|  |  | Baseline Bias | 0.253 | 0.206 | 0.027 | 7.560 | <.001 |
|  |  | C_Unemploy | 0.021 | 0.001 | 0.001 | 0.718 | .473 |
|  |  |  |  |  |  |  |  |
|  | Model 7 | Move-In Bias | 0.285 | 0.752 | 0.096 | 7.826 | <.001 |
|  |  | Baseline Bias | 0.260 | 0.214 | 0.030 | 7.083 | <.001 |
|  |  | M_Income | 0.052 | 0.000 | 0.000 | 1.636 | 0.102 |
|  |  |  |  |  |  |  |  |
|  | Model 8 | Move-In Bias | 0.285 | 0.753 | 0.096 | 7.855 | <.001 |
|  |  | Baseline Bias | 0.255 | 0.210 | 0.03 | 6.909 | <.001 |
|  |  | C_Income | 0.070 | 0.001 | 0.001 | 2.194 | .029* |
|  |  |  |  |  |  |  |  |
| 2016-2020 | Model 5 | Move-In Bias | 0.420 | 0.997 | 0.068 | 14.722 | <.001 |
|  |  | Baseline Bias | 0.259 | 0.217 | 0.024 | 9.158 | <.001 |
|  |  | M_Unemploy | -0.034 | -0.002 | 0.001 | -1.264 | .206 |
|  |  |  |  |  |  |  |  |
|  | Model 6 | Move-In Bias | 0.424 | 1.007 | 0.067 | 15.011 | <.001 |
|  |  | Baseline Bias | 0.260 | 0.218 | 0.024 | 9.212 | <.001 |
|  |  | C_Unemploy | -0.023 | -0.001 | 0.001 | -0.859 | .039* |
|  |  |  |  |  |  |  |  |
|  | Model 7 | Move-In Bias | 0.427 | 1.023 | 0.074 | 13.864 | <.001 |
|  |  | Baseline Bias | 0.259 | 0.215 | 0.025 | 8.526 | <.001 |
|  |  | M_Income | 0.069 | 0.000 | 0.000 | 2.394 | 0.017* |
|  |  |  |  |  |  |  |  |
|  | Model 8 | Move-In Bias | 0.418 | 1.000 | 0.073 | 13.627 | <.001 |
|  |  | Baseline Bias | 0.266 | 0.220 | 0.025 | 8.736 | <.001 |
|  |  | C_Income | 0.029 | 0.000 | 0.000 | 1.028 | 0.304 |

**Table S12 Model Results for Socioeconomic Changes and Explicit Bias**

|  |  | **Predictor** | ***B*** | **β** | ***SE*** | ***t*** | ***p*** |
| --- | --- | --- | --- | --- | --- | --- | --- |
| 2006-2010 | Model 5 | Move-In Bias | 0.357 | 1.137 | 0.104 | 10.964 | <.001 |
|  |  | Baseline Bias | 0.255 | 0.281 | 0.037 | 7.637 | <.001 |
|  |  | M_Unemploy | -0.073 | -0.010 | 0.004 | -2.243 | .025* |
|  |  |  |  |  |  |  |  |
|  | Model 6 | Move-In Bias | 0.377 | 1.166 | 0.104 | 11.188 | <.001 |
|  |  | Baseline Bias | 0.259 | 0.286 | 0.037 | 7.745 | <.001 |
|  |  | C_Unemploy | 0.026 | 0.003 | 0.004 | 0.803 | .423 |
|  |  |  |  |  |  |  |  |
|  | Model 7 | Move-In Bias | 0.415 | 1.219 | 0.102 | 11.985 | <.001 |
|  |  | Baseline Bias | 0.281 | 0.307 | 0.037 | 8.168 | <.001 |
|  |  | M_Income | 0.146 | 0.004 | 0.001 | 4.350 | <.001*** |
|  |  |  |  |  |  |  |  |
|  | Model 8 | Move-In Bias | 0.389 | 1.143 | 0.102 | 11.171 | <.001 |
|  |  | Baseline Bias | 0.287 | 0.314 | 0.038 | 8.277 | <.001 |
|  |  | C_Income | 0.080 | 0.006 | 0.002 | 2.379 | 0.018* |
|  |  |  |  |  |  |  |  |
| 2011-2015 | Model 5 | Move-In Bias | 0.192 | 0.658 | 0.113 | 5.823 | <.001 |
|  |  | Baseline Bias | 0.367 | 0.378 | 0.034 | 11.066 | <.001 |
|  |  | M_Unemploy | -0.059 | -0.008 | 0.004 | -1.953 | .051 |
|  |  |  |  |  |  |  |  |
|  | Model 6 | Move-In Bias | 0.201 | 0.688 | 0.112 | 6.132 | <.001 |
|  |  | Baseline Bias | 0.378 | 0.390 | 0.034 | 11.514 | <.001 |
|  |  | C_Unemploy | -0.004 | -0.001 | 0.004 | -0.140 | .889 |
|  |  |  |  |  |  |  |  |
|  | Model 7 | Move-In Bias | 0.198 | 0.649 | 0.117 | 5.553 | <.001 |
|  |  | Baseline Bias | 0.393 | 0.397 | 0.037 | 10.824 | <.001 |
|  |  | M_Income | -0.007 | -0.000 | 0.001 | -0.232 | 0.817 |
|  |  |  |  |  |  |  |  |
|  | Model 8 | Move-In Bias | 0.201 | 0.658 | 0.116 | 5.699 | <.001 |
|  |  | Baseline Bias | 0.387 | 0.391 | 0.037 | 10.708 | <.001 |
|  |  | C_Income | 0.016 | 0.001 | 0.003 | 0.497 | 0.620 |
|  |  |  |  |  |  |  |  |
| 2016-2020 | Model 5 | Move-In Bias | 0.495 | 1.075 | 0.053 | 20.310 | <.001 |
|  |  | Baseline Bias | 0.322 | 0.332 | 0.025 | 13.397 | <.001 |
|  |  | M_Unemploy | -0.130 | -0.019 | 0.003 | -5.834 | <.001*** |
|  |  |  |  |  |  |  |  |
|  | Model 6 | Move-In Bias | 0.517 | 1.122 | 0.053 | 21.167 | <.001 |
|  |  | Baseline Bias | 0.339 | 0.349 | 0.025 | 13.848 | <.001 |
|  |  | C_Unemploy | 0.045 | 0.008 | 0.004 | 2.052 | .040* |
|  |  |  |  |  |  |  |  |
|  | Model 7 | Move-In Bias | 0.567 | 1.220 | 0.054 | 22.645 | <.001 |
|  |  | Baseline Bias | 0.311 | 0.323 | 0.026 | 12.534 | <.001 |
|  |  | M_Income | 0.167 | 0.003 | 0.000 | 7.405 | <.001*** |
|  |  |  |  |  |  |  |  |
|  | Model 8 | Move-In Bias | 0.553 | 1.190 | 0.054 | 21.903 | <.001 |
|  |  | Baseline Bias | 0.319 | 0.331 | 0.026 | 12.677 | <.001 |
|  |  | C_Income | 0.122 | 5.364 | 0.001 | 5.364 | <.001*** |

1. We did not use GDP because the Bureau of Economic Analysis has stopped offering public access to county-level GDP earlier than 2017. To ensure we have a complete analysis on all three time periods, we decided to use county-level personal income instead. [↑](#footnote-ref-2)
